# Cross-sectional imaging of iatrogenic complications after extracorporeal and endourological treatment of urolithiasis

**DOI:** 10.1007/s13244-014-0355-z

**Published:** 2014-09-26

**Authors:** Massimo Tonolini, Federica Villa, Sonia Ippolito, Alessandra Pagani, Roberto Bianco

**Affiliations:** Department of Radiology, “Luigi Sacco” University Hospital, Via G.B. Grassi 74, 20157 Milan, Italy

**Keywords:** Urolithiasis, Lithotripsy, Ureteroscopy, Complications, Computed Tomography (CT)

## Abstract

Extracorporeal shock wave lithotripsy (ESWL), percutaneous nephrolithotomy (PCNL) and ureteroscopy (URS) currently represent the mainstay treatment options for the vast majority of patients with urolithiasis, with limited contraindications and high success rates. However, minimally invasive extracorporeal and endourological treatments are associated with a non-negligible morbidity including occasional life-threatening occurrences. These complications represent a source of concern for urologists since they may result in prolonged hospitalisation, need for surgical, endoscopic or interventional treatment, long-term renal impairment, and sometimes even medical malpractice claims. Due to the increasing prevalence of urolithiasis and the large number of therapeutic procedures performed, in hospitals with active urologic practices radiologists are increasingly requested to investigate suspected post-procedural complications following ESWL, PCNL or ureteroscopic stone removal. Based upon our experience, this pictorial essay provides an overview of current extracorporeal and endourological treatment modalities for urolithiasis, including indications and possible complications according to the most recent guidelines from the European Association of Urology (EAU). Afterwards, we review the clinical features and cross-sectional imaging appearances of common and unusual complications with case examples, including steinstrasse, subcapsular, perirenal and suburothelial haemorrhages, severe urinary tract infections (such as pyeloureteritis, pyelonephritis, renal abscesses and pyonephrosis), ureteral injuries and delayed strictures.

*Teaching points*

• *Extracorporeal lithotripsy, percutaneous nephrolitotomy and ureteroscopy allow treating urolithiasis.*

• *Minimally invasive extracorporeal and endourological treatment have non-negligible morbidity.*

• *Multidetector CT allows confident assessment of stone-free status and postprocedural complications.*

• *Main complications include steinstrasse, bleeding, severe infections, ureteral injuries and strictures.*

• *Imaging triage allows the choice among conservative, surgical, endoscopic or interventive treatment.*

## Introduction

### Background

Urolithiasis represents one of the most common urogenital disorders and occurs in approximately 4–5 % of the general population in European countries. In the USA, the lifetime risk of symptomatic kidney stones has been estimated to approximate 13 % in men and 7 % in women, leading to substantial cost in terms of emergency department visits, use of imaging and treatment. The increasing prevalence and incidence of urolithiasis reported over the last decades probably results from the combined effects of nutritional changes, environmental factors and improved diagnosis, particularly with the extensive use of unenhanced multidetector CT (MDCT) [1, 2].

During the last 30 years, modern extracorporeal and endourological therapies have revolutionised the field of urology and dramatically reduced the number of surgical procedures, which are now second- or third-line treatment options reserved for only 1–1.5 % of patients. Extracorporeal shock wave lithotripsy (ESWL), percutaneous nephrolithotomy (PCNL) and retrograde ureteroscopy (URS) currently represent the established treatment for the vast majority of urolithiasis cases needing active stone removal [3, 4].

However, despite increasing operator experience and technical advancements including refined energy applications and endourological equipment, minimally invasive therapies for urolithiasis are associated with a non-negligible morbidity. Iatrogenic complications represent a source of concern for urologists since they may require prolonged hospitalisation and additional surgical, endoscopic or interventional treatment, may have detrimental effects on renal function and sometimes even lead to medical malpractice claims [4–6].

### Aim

Most previous reports in the radiological literature mainly focussed on imaging complications after nephrostomy and ureteral stenting using conventional radiographic techniques such as plain radiographs and trans-nephrostomy pyelography [7, 8].

Although improved techniques, experience and instrumentation have led to a decreasing incidence of urologic iatrogenic injuries during the past decade, due to the large number of urological procedures performed, radiologists are increasingly requested to investigate suspected early or delayed complications following ESWL, PCNL or ureteroscopic stone removal [5]. Based upon our experience, this article discusses the fundamentals and indications of minimally invasive treatment options for urolithiasis, then reviews and illustrates the clinical features and cross-sectional imaging appearances of common and unusual complications with emphasis on MDCT, aiming to provide radiologists with an increased familiarity with post-procedural imaging in patients being treated for urolithiasis.

## Minimally invasive treatment of urolithiasis

### Overview and indications

According to the most recent guidelines from the European Association of Urology (EAU), the main indications for active removal of renal calculi include stone growth, obstruction, associated infection, renal insufficiency, and acute and/or chronic pain. Nowadays, open or laparoscopic surgery is reserved for the few patients with complex stone burden after failure of ESWL or endourological procedures, morbid obesity, anatomical abnormalities (such as renal ectopia) not amenable to other therapies and non-functioning kidneys requiring nephrectomy [4].

Currently, there is a consensus that most complex (including partial and complete staghorn) stones should be primarily approached by means of PCNL. ESWL represents the preferred initial approach for pelvic, upper or mid-calyceal calculi up to 2 cm in size as well as for sub-centimetre distal ureteral stones including those at the vesico-ureteral junction. Conversely, due to the limited efficacy of ESWL, endourological procedures such as PCNL and retrograde intrarenal surgery (RIRS) are recommended for large (>2 cm), shockwave-resistant (such as those containing calcium oxalate) or lower pole renal stones. Finally, URS represents the ideal modality for both multiple or single ureteral stones above 1 cm [4].

### Extracorporeal shock wave lithotripsy

Since its introduction in the early 1980s, ESWL has dramatically changed the management of urolithiasis and is currently accepted as the preferred and least invasive first-line treatment for the majority (almost 90 %) of patients with renal and ureteral calculi without spontaneous passage. During the last 30 years, its use has become widespread, and millions of treatments have been performed worldwide. Its limited contraindications including pregnancy, coagulation disorders, cardiac arrhythmias, uncontrolled urinary infection, renal failure, anatomic urinary tract abnormalities or obstruction distal to the stone, severe skeletal deformity and obesity, and proximity to an arterial aneurysm [4, 9, 10].

During ESWL, shock waves generated outside the body cross through the soft tissues without loss of strength and cause disintegration of urinary stones into smaller portions through direct shearing force, erosion or cavitation. With the increasing availability of newer and improved lithotripters, the patient tolerance and efficacy of the procedure have increased; therefore, ESWL is usually performed in an outpatient setting without general anaesthesia and can be repeated as necessary. Routine pre-treatment stenting has now been abandoned because it contributes minimally to the prophylaxis of complications and may reduce the stone-free rate (SFR). ESWL achieves a 78–82 % overall success rate (SFR at 3 months) with a significant (45–53 %) proportion of repeated treatments and 8.4–11 % need for auxiliary procedures [4, 9–14].

Although it may seem a noninvasive modality, ESWL has a reported 15.3 % overall complications rate, which is inferior to that occurring after PCNL and URS. The most common occurrences include cardiac dysrhythmia (11–5 %), bacteriuria (7.7–23 %), bleeding (4–19 %), steinstrasse (3.6–7 %), renal colic (2–4 %) and urosepsis (1–2.7 %) in descending order of frequency, plus sporadic severe cardiovascular events, bowel perforations, liver or spleen haematomas. However, major post-ESWL complications are very uncommon compared to the vast number of procedures performed worldwide and include mostly steinstrasse, severe haemorrhage and urosepsis [4, 13, 14].

Although the mechanism underlying haemorrhage involves the compressive and tensile force of the shock waves on the soft tissues coupled with the cavitation effect on cell integrity, no clear correlation exists with the number and intensity of applied shock waves. Predisposing factors include hypertension, diabetes, obesity, advanced age, coagulation deficits and use of antiplatelet medications. Symptomatic or clinically significant bleeding requiring blood replacement occurs in approximately 2 out of 1,000 treated patients. Similarly, a large multicentre study assessed the incidence of renal haematomas at 0.5 and 0.14 % following ESWL treatment of renal and ureteral stones respectively [4, 9–11, 13–16]. Retroperitoneal haemorrhage after ESWL may cause haemodynamic shock and occasionally be fatal [17, 18].

### Percutaneous nephrolithotomy

Currently considered the standard procedure for large renal calculi, PCNL is increasingly performed without nephrostomy or ureteral stenting and with the use of ultrasound, pneumatic and Ho:YAG laser devices as well as forceps or nitinol baskets for stone extraction. Contraindications include untreated infections, pregnancy, atypical bowel interposition, and known or suspected kidney tumour. Compared to ESWL, PCNL achieves stone-free status in 95 and 85 % of cases at 1 week and 3 months, respectively, without the need for repeated procedures in the vast majority of cases [4, 19].

Although most patients experience an uneventful postoperative course, percutaneous treatment of urolithiasis is associated with 20–29 % overall and 4–5.2 % severe complication rates, and occasional fatalities. High-risk patients include those with anatomical abnormalities, large or staghorn stones, and comorbidities, such as diabetes, and those requiring upper pole or multisite access. Pre-existent urine infection and absent hydronephrosis represent risk factors for sepsis and severe bleeding, respectively. Following PCNL the most common adverse events include fever (10–23 %) and urinary infection, respiratory impairment (due to pneumothorax, pleural effusion, atelectasis or pneumonia), ileus, bleeding, urosepsis (0.5 % of patients), renal pelvis laceration and ureteral stricture, in descending order of frequency, plus sporadic cases of visceral injuries to the spleen and large bowel. Surgical or interventional treatment is required in up to one-third of all complications [4, 5, 20–27].

One of the most common and worrisome PCNL-related complications, haemorrhage, occurs in variable entities after nearly one-third of procedures. Perioperative bleeding requiring blood transfusions and/or operative treatment is reported in 3.8–11 % of cases, particularly in patients with comorbidities, advanced age, staghorn calculi and prolonged operative time. After PCNL bleeding may be venous, acute (from injury to the anterior or posterior segmental arteries) or delayed (due to interlobar and lower-pole arterial lesions, arterio-venous fistula or post-traumatic aneurysm) [5, 20, 21, 23, 25, 27, 28].

### Ureteroscopic stone removal

URS represents an established minimally invasive treatment with a high success rate and acceptable morbidity, which has dramatically improved the outcome of patients with ureteral calculi. Increasingly performed without routine ureteral stenting, URS has recently undergone technical advances including improved equipment, which allows RIRS, intracorporeal lithotripsy using the Ho:YAG laser, and complete stone removal using endoscopic forceps or nitinol baskets. As a result, the expanded indications now range from treatment of small distal ureter stones using a semirigid URS to larger sized renal pelvis stones treated by a flexible URS [4, 29].

Compared to ESWL, URS offers a superior success rate (approximately 90 %) at the expense of greater invasiveness with increased morbidity and longer hospital stays. Reported results include 81.9 %, 87.3 % and 94.9 % SFR for the proximal, mid- and distal ureter respectively, a 7.75 % retreatment rate and need for auxiliary procedures in 18.6 % of cases [14, 30].

The majority of postoperative URS complications are minor occurrences such as haematuria and urinary infection. The risk is further increased by preoperative bacteriuria, hydronephrosis, longer operative time and limited hospital experience. More significant occurrences include persistent haematuria (2 % of cases), renal colic (2.2 %), urosepsis (1.1 %) and bleeding (0.1 %). Furthermore, aggressive ureteroscopic procedures such as RIRS currently represent the leading cause of the reported increase in iatrogenic ureteral injuries. Whereas mucosal damage is relatively common (41–46 % of URS procedures), severe injury involving the muscular layer or deeper may occur in up to 13–18 % of cases and may be largely reduced by prophylactic stenting. Recognition and treatment of ureteral injuries at the time of endourological procedures are strongly related to a better outcome. Ureteral perforation, avulsion or lost segment represent the rarest yet dreaded occurrences [4, 31–35].

## Clinical setting and indications for post-procedural imaging

**Fig. 1:**
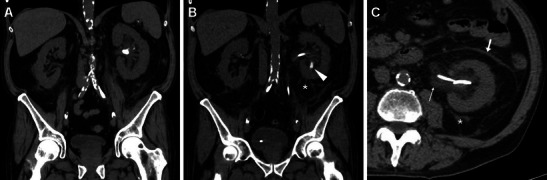
Usual early post-procedural imaging appearances following extracorporeal shock wave lithotripsy (ESWL) in a 51-year-old male with a 12-mm stone in the left renal pelvis detected by preoperative unenhanced multidetector CT (MDCT, A). Twenty-four hours after ESWL, repeat MDCT (B, C) was requested because persistent flank pain showed a ureteral stent in place, residual stone fragment in the lower pole calyx (*arrowhead in B*), minimal perirenal fluid (*) and ipsilateral fascial effusion (arrow in C), and thickened urothelium of the renal pelvis (*thin arrow in C*). Afterwards, the patient experienced an uneventful postoperative course

**Fig. 2:**
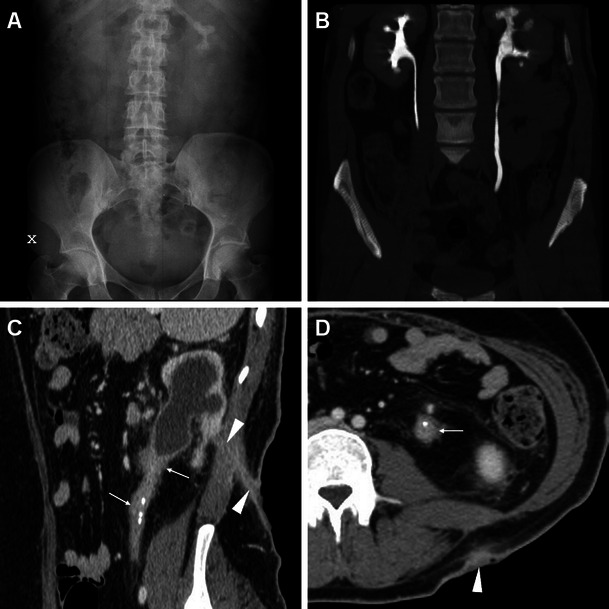
A 45-year-old female with diabetes, antiretroviral-treated human immunodeficiency virus (HIV) infection and left partial staghorn nephrolithiasis at previous plain radiograph (**A**) and MDCT urography (**B**) presented 9 weeks after percutaneous nephrolithotomy (PCNL) with clinical and contrast-enhanced MDCT (**C**, **D**) evidence of nephrocutaneous fistulisation reaching a cutaneous orifice (*arrowheads*). Associated findings included ipsilateral hydronephrosis with parenchymal thinning, severely thickened hyperenhancing urothelium (*thin arrows*) in the ureteropelvic junction, proximal and mid-ureter, and multiple residual stone fragments along the lumbar ureter consistent with steinstrasse. Surgical treatment included nephro-ureterectomy with fistula debridement. (Partly reprinted with permission from [51])

In most patients, clinical suspicion of early iatrogenic complications is based on a combination of intraoperative findings with flank pain, persistent haematuria, fever, clinical or laboratory evidence of blood loss and/or systemic inflammation hours or days after the procedure, prompting the urologic surgeon to request urgent imaging. The majority of post-procedural complications after ESWL and endourological procedures are minor and result from urine infection or passage of small stone fragments along the ureter. However, since pain, dysuria and transient haematuria are common complaints reported in up to 40 % of patients, diagnostic imaging often proves crucial in the correct assessment of treated patients, particularly to detect and quantify post-procedural bleeding. As already mentioned, at some institutions noncontrast MDCT is routinely obtained to assess the post-procedural stone-free status. Post-treatment unenhanced MDCT is extremely sensitive for detecting residual lithiasis (Fig. 1) and commonly shows mild perirenal and fascial fluid or blood that does not require treatment or prolonged hospitalisation (Fig. 1). The characteristic “steinstrasse” appearance with calculi lining up along the ureter (Fig. 2) results from stone fragmentation and distal migration and represents an indication for URS. Furthermore, interpretation of unenhanced MDCT provides important information concerning possible complications, including excellent sensitivity for detection of ipsilateral basal pleuropulmonary changes, persistent hydronephrosis, fascial and paracolic gutter fluid, and perinephric fluid or blood [3, 10, 27, 36].

With significant clinical, laboratory or unenhanced MDCT findings, further investigation with iodinated contrast medium (CM) is warranted unless contraindicated by renal failure. Although ultrasound may rapidly detect most abnormal collections, similarly to the trauma setting, enhanced multiphase MDCT comprehensively provides accurate detection and classification of iatrogenic lesions of the kidney and ureter, which are crucial for optimal treatment choice and successful patient management. After a preliminary unenhanced acquisition to detect hyperattenuating blood, arterial-dominant and venous-phase images after intravenous CM injection are recommended, particularly to identify CM extravasation indicating active bleeding. Finally, excretory phase imaging obtained at least 5–20 min later allows visualising the opacified collecting systems and detecting iodinated urine leaks and urinomas. In order to limit the ionising radiation exposure in younger patients, at most institutions classical multiphase MDCT is being increasingly replaced by time- and dose-efficient split-bolus acquisitions such as the triple-bolus MDCT urography protocol, which provides simultaneous renovascular, corticomedullary, nephrographic and excretory imaging. Furthermore, repeated MDCT provides consistent monitoring of injuries after conservative or interventional treatment [37–42].

Similarly to post-surgical injuries, iatrogenic trauma to the ureter from endourological procedures is often (in almost two-thirds of cases) undetected intraoperatively. Unfortunately, delayed diagnosis is associated with a worse outcome due to superinfection and renal damage. Clinically, ureteral injury may be suggested by flank pain, urinary incontinence, vaginal or drain urinary leakage, haematuria, fever, worsening renal function, or imaging detection of hydronephrosis or urinoma [5, 33, 34]. MDCT urography and—in selected patients—MR urography represent the cross-sectional techniques of choice to investigate suspected ureteral lesions noninvasively. Unenhanced heavily T2-weighted MR sequences allow visualisation of the static fluid content of the urinary tract, are most useful with dilated or obstructed collecting systems, and may be completed by means of gadolinium-enhanced excretory MR urography as needed [43].

## Cross-sectional imaging appearances of iatrogenic injuries

### Haemorrhages

**Fig. 3:**
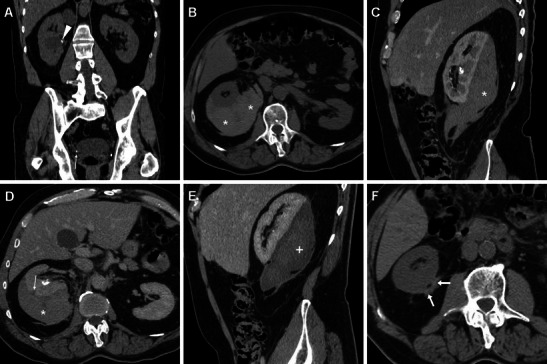
A 63-year-old male patient with comorbidities suffered from syncope, flank pain and tenderness, with a 3 g/dl haemoglobin drop 6 h after ESWL treatment of right-sided lithiasis of the renal pelvis (*arrowhead*) and distal ureter (*thin arrows*) at preoperative unenhanced MDCT (A). After bedside ultrasound (not shown) detected an inhomogeneously echoic renal collection, urgent multiphase contrast-enhanced MDCT including multiplanar reformatted images (B, C, D) confirmed a hyperattenuating (55–60 HU) acute subcapsular renal haematoma (*) extending towards the diaphragmatic crus. The ventrally displaced right kidney appeared functioning with residual lithiasis and a focal parenchymal laceration (thin arrow in D) in the nephrographic acquisition. Atelectasis and moderate pleural effusion were seen at the ipsilateral lung base. Hydronephrosis, active bleeding and urine extravasation were excluded. Conservative treatment including blood transfusions achieved clinical improvement and stabilisation of the haematocrit. Follow-up MDCT 3 weeks later (E) showed haematoma (*) with decreased attenuation (35–40 HU) indicating initial liquefaction. One year later, unenhanced MDCT during another episode of renal colic demonstrated resolved haematoma (arrows in F)

**Fig. 4:**
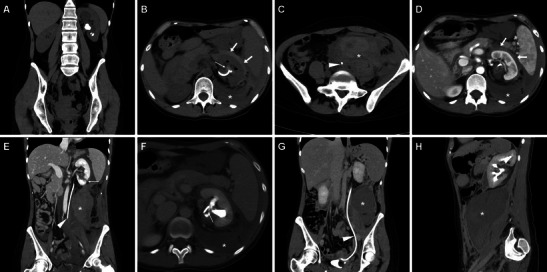
A 33-year-old female with nephrolithiasis and previous unsuccessful ESWL treatment 3 years earlier underwent PCNL with ureteral stenting. Preoperative unenhanced MDCT (A) showed the left kidney with moderate pelvicalyceal dilatation, a 2-cm renal pelvis stone plus two additional 8–9 mm lower calyx stones. Intractable flank and abdominal pain with laboratory signs of blood loss (6.2 mg/dl nadir haemoglobin) led to urgent contrast-enhanced multiphase MDCT hours after the procedure. MDCT (B–F) detected a fresh clot in the left pelvicalyceal system (*thin arrows in B and F*), thin subcapsular haematoma (*arrows*), massive perirenal and posterior pararenal haemorrhage (*) causing anterior displacement of spleen and kidney plus ureter medialisation (*arrowheads*). A focal injury at the lower renal pole (thin arrow in E) corresponded to the instrumentation access site, without signs of active bleeding and extravasated urine. Urologists opted for conservative treatment including blood transfusions. Seven days later, with improving clinical conditions, repeat MDCT (G, H) showed retroperitoneal haemorrhage (*) with well-demarcated margins and decreasing size, attenuation values and mass effect (*arrowhead in G*)

**Fig. 5:**
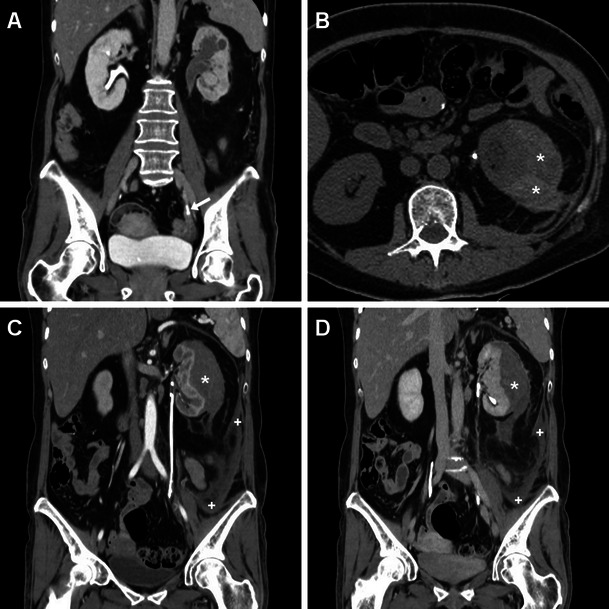
A 55-year-old female with a 12-mm calculus in the left pelvic ureter (*arrow in A*) suffered fever and anaemisation (7.8 g/dl haemoglobin) 48 h after ureteroscopic lithotripsy. Unenhanced (B) and multiphase contrast-enhanced (C, D) MDCT detected a sizeable subcapsular haematoma (*) causing moderate parenchymal compression and associated with blood effusion along the ipsilateral fasciae (+), without signs of active bleeding. Note the ureteral stent left in place intraoperatively. Conservative treatment included blood transfusions and prolonged hospitalisation

**Fig. 6:**
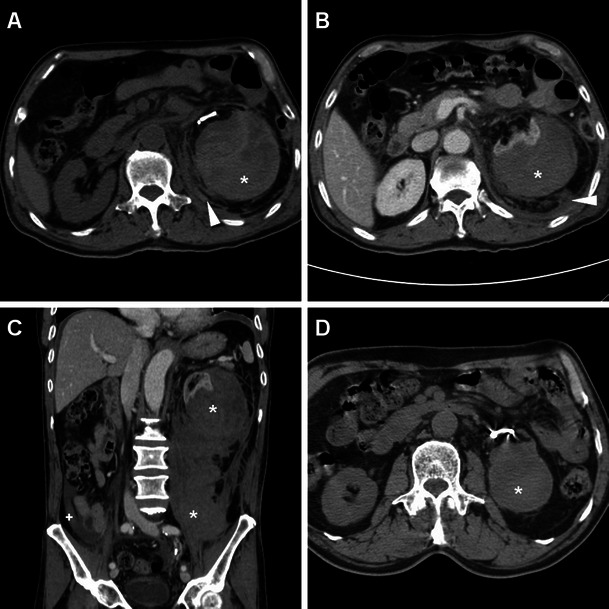
A 66-year-old male experienced severe flank pain with acute blood loss shortly after ureteroscopic stone removal. Unenhanced (A), arterial (B) and venous (C) phase MDCT images showed large subcapsular renal haematoma (*) with extensive retroperitoneal bleeding including the posterior pararenal space (*arrowheads*), causing severe parenchymal compression. Contrast medium and urine extravasation were excluded, thus allowing conservative treatment. A month later follow-up unenhanced MDCT (D) with the ureteral stent still in place showed well-demarcated residual haematoma with decreased size and attenuation values (*)

**Fig. 7:**
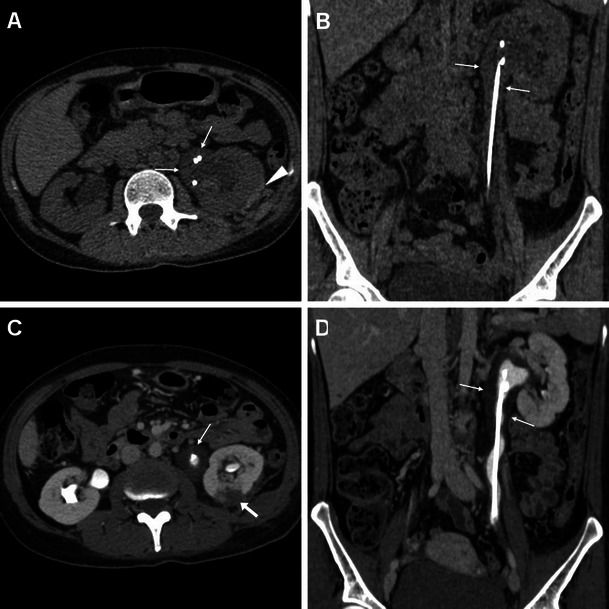
After PCNL treatment of a 2-cm left renal pelvis stone, a 46-year-old female was not discharged because of a progressive, asymptomatic haemoglobin drop (nadir 8.4 g/dl). Four days later, unenhanced MDCT showed a ureteral stent in place, hyperattenuating (50 HU) circumferential mural thickening of the renal pelvis and proximal ureter (*thin arrows in A, B*) consistent with suburothelial haemorrhage, and minimal blood in the ipsilateral perirenal and posterior pararenal spaces (*arrowhead in A*). Study completion with MDCT urography showed a functioning left kidney with a 2-cm devascularised injury (arrow in C) in the dorsal middle third and hypodense suburothelial haemorrhage (*thin arrow in C, D*) compared to the well-opacified collecting system. Conservative management including blood transfusions allowed hospital discharge in a few days and normalisation of the clinical, biochemical and imaging abnormalities within a month

Minimal or moderate degrees of perinephric blood are observed in approximately one-third of patients studied with MDCT shortly after ESWL and PCNL, most usually appearing as dense thickening of the perirenal septa (Fig. 1) [27, 36]. Conversely, clinically significant iatrogenic haematomas appear as hyperattenuating collections (45 to 90 Hounsfield Units, HU) on precontrast scans depending on their more or less acute stage, being relatively hyperdense compared to the renal parenchyma (Figs. 3, 4, 5 and 6). In our experience, iatrogenic haematomas are most commonly or initially subcapsular with the characteristic crescent- or biconvex-shaped appearance limited by the renal capsule, causing compression on the adjacent parenchyma, indenting or flattening the renal margin. Haemorrhage extends variably into the retroperitoneum, mostly by occupying the peri- and pararenal spaces. Primarily perinephric haematomas are demarcated by the Gerota’s fascia and cause renal displacement. Furthermore, focal parenchymal injuries closely similar to traumatic lacerations may be observed as non-enhancing linear or irregular clefts (Figs. 3, 4 and 7). Heralded by linear or flame-shaped high attenuation (85—370 HU) structures corresponding to CM extravasation, active bleeding at MDCT indicates probable failure or nonsurgical management and represents the strongest indication for interventional or surgical treatment. Furthermore, MDCT provides consistent follow-up of conservatively treated lesions showing progressive demarcation, size reduction and decreasing attenuation of haematomas (Figs. 3, 4 and 6) [16, 39].

The majority of iatrogenic renal haematomas tend to reabsorb and can be successfully managed conservatively, including appropriate transfusion support when signs of hypovolemic shock or haemoglobin decrease are present. Conversely, angiographic embolisation or surgical exploration are reserved for haematomas with MDCT evidence of ongoing arterial bleeding and for those cases that do not respond to a “watchful waiting” strategy. Required in less than 1 % of patients, superselective embolisation is the treatment of choice and extremely effective in stopping haemorrhage, with 100 % technical and 95 % clinical success rates [5, 16, 28, 44, 45].

Most haematomas resolve within 2 years without adverse effects on blood pressure and renal function. Persisting liquefied haematomas may be amenable to percutaneous drainage. Alternatively, the “Page kidney” phenomenon consisting of long-term development of hypertension from renal compression, ischaemia and hypoperfusion causing activation of the renin-angiotensin-aldosterone system has been reported [9, 46].

Furthermore, after PCNL, bleeding may occasionally manifest as suburothelial haemorrhage (SH) of the renal pelvis and ureter (Fig. 7). A very rare although well-established condition, spontaneous SH most usually occurs secondary to an excessive warfarin anticoagulant therapy condition, sometimes with haemophilia or other coagulation disorders. The characteristic CT appearance includes a circumferential hyperattenuating mural thickening of the renal pelvis, sometimes extending along the proximal ureter, with an increased density that is often subtle and better appreciable on unenhanced scans with narrow window settings. On nephrographic images, the mural thickening is less conspicuous and appears as soft-tissue thickening, hypodense compared to opacified urine in the collecting system. Despite encasement of the renal infundibulum, luminal compression and upstream calyceal dilatation are absent or minimal. In the excretory phase small nodular filling defects or predominantly smooth narrowing of the renal pelvis and ureter are noted, so that misinterpretation as pyeloureteritis cystica, pyonephrosis or transitional cell carcinoma may occur. The usual fate is rapid, complete resolution of imaging changes following conservative treatment [47–49].

### Severe urinary tract infections

**Fig. 8:**
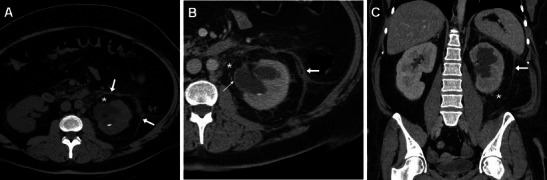
Three days after PCNL treatment, a 40-year-old female underwent contrast-enhanced MDCT because of septic fever. Unenhanced (A) and contrast-enhanced (B, C) images showed left-sided pelvicalyceal dilatation with inflammatory-type stranding of the surrounding fat (*), mild enhancing urothelial thickening (thin arrow in B), ipsilateral fascial effusion (arrows) and decreased nephrographic parenchymal enhancement compared to the contralateral kidney (C). Hydronephrosis was due to small residual stone fragments in the lumbar ureter (not shown). Clinical and imaging suspicion of pyonephrosis was confirmed and relieved by positioning of the ureteral stent plus intensive antibiotic therapy

**Fig. 9:**
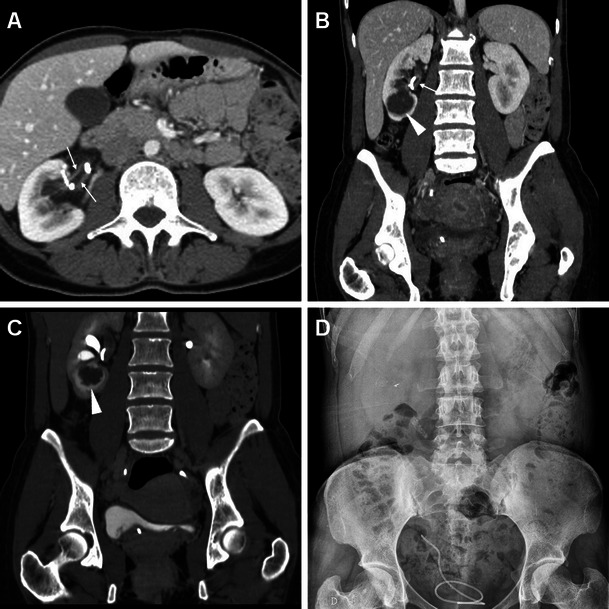
A 38-year-old female with intermittent fever and elevated C-reactive protein 2 weeks after PCNL treatment for right-sided nephrolithiasis. Contrast-enhanced MDCT with the ureteral stent in place showed absent hydronephrosis, mild enhancing ureteral thickening (*thin arrows in A, B*) indicating infectious pyeloureteritis and a 3-cm hypoattenuating lesion with peripheral delayed enhancement (*arrowheads in B and C*) at the lower renal pole consistent with an abscess. The patient recovered after intensive antibiotic treatment. During follow-up, distal stent migration was detected radiographically (D)

Cross-sectional imaging signs of urinary infection are mostly conspicuous in the nephrographic phase MDCT acquisition. Infectious pyeloureteritis is heralded by pelvicalyceal and/or ureteral thickening with prominent urothelial enhancement (Figs. 2, 8 and 9). Because of inflammatory debris obstructing the renal tubules and impaired function from tubular ischaemia, acute pyelonephritis appears as a more or less enlarged, oedematous kidney with decreased parenchymal enhancement (Fig. 8) or with the characteristic “striated nephrogram” appearance including well-defined wedge-shaped areas, linear bands or streaky zones of reduced enhancement perpendicular to the cortical surface. Accessory signs such as perinephric fat stranding and thickening of Gerota’s fascia are commonly observed (Fig. 8). Progression of infection may lead to the formation of variable-sized intra- or perirenal abscesses (Fig. 9) with a characteristic appearance of round or geographic hypoattenuating collections, usually surrounded by a peripheral thickened enhancing capsule and by decreased parenchymal enhancement corresponding to non-necrotic infected kidney [50, 51].

Defined by an infected hydronephrosis, pyonephrosis represents a urological emergency that requires prompt intervention to prevent sepsis and development of impaired renal function. Radiologists should maintain a high level of suspicion, since imaging differentiation of pyonephrosis from hydronephrosis is challenging. Useful signs of an infected collecting system include renal enlargement with a striated or poorly functioning nephrogram, perinephric fat inflammatory changes and urothelial thickening (Fig. 8). Occasionally the obstructed cavities show higher-than-water (10–50 HU) attenuation and/or fluid-fluid levels corresponding to purulent urine. Decompression by means of nephrostomy or ureteral stenting relieves obstruction and allows confirming the diagnosis [50, 51].

### Ureteral injuries and urinomas

Major ureteral injuries with full-thickness mural involvement are increasingly reported after ureteroscopic stone removal (including RIRS) and occasionally PCNL, and involve the proximal, middle and distal ureter in 51 %, 14 % and 35 % of cases, respectively. Resulting from disruption of the urinary tract, iatrogenic urinomas represent abnormal collections of extravasated urine, which may collect close to the site of the renal pelvis or ureteral injury and otherwise variably dissect into the retroperitoneum. Often clinically unsuspected but associated with significant morbidity from superinfection, iatrogenic urinomas are mostly treated by prolonged nephrostomy or ureteral stenting [35, 37, 38].

Whereas in the past ureteral lacerations and urinomas were usually depicted by means of trans-nephrostomy or ascending pyelography, nowadays the diagnosis is usually made by MDCT. Urinomas commonly appear as confined collections measuring 0–20 HU in unenhanced scans and may be misinterpreted as ascites, abdominal or pelvic abscesses, cystic masses or chronic liquefied haematomas. The imaging hallmark of the urinoma is represented by opacification (up to 80–200 HU) corresponding to an enhanced urine leak observed on excretory-phase or MDCT urography acquisitions. Opacification is usually inhomogeneous in larger lesions and usually progresses over repeated delayed-phase acquisitions [37–40].

**Fig. 10:**
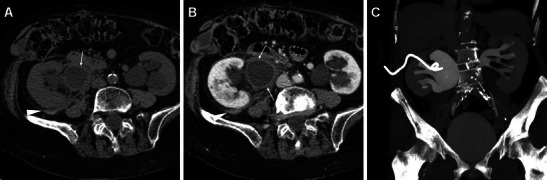
One day after PCNL treatment of right-sided urolithiasis, unenhanced (A) and contrast-enhanced (B) MDCT obtained in a 75-year-old female revealed mild uniform thickening of the pelvis (*thin arrows*) consistent with suburothelial haemorrhage and/or inflammation, plus minimal posterior perirenal blood (*arrowheads*), which did not require therapy. Two years later, a coronal MIP reformatted image from MDCT urography (C), requested because of worsening renal function, showed persistent dilatation of the renal pelvis due to iatrogenic stricture of the ureteropelvic junction, with preserved parenchymal function and collecting system opacification, which was treated with long-term nephrostomy

**Fig. 11:**
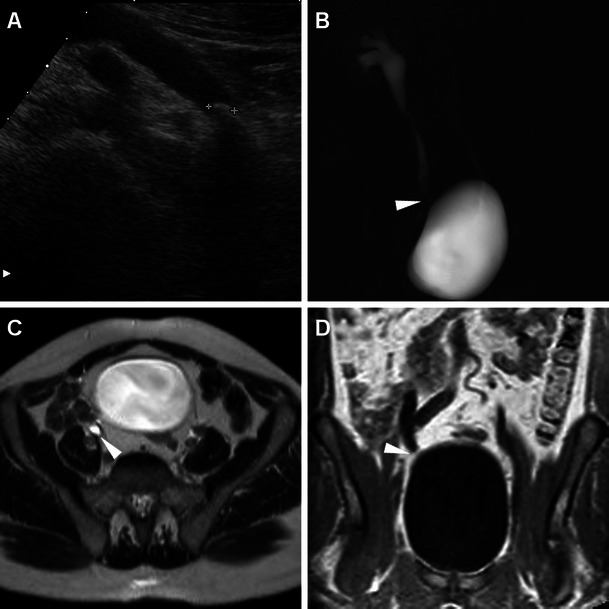
A 51-year-old male received ureteroscopic removal of a distal ureteral calculus (*ultrasound in A*). Fifteen months later, persistent upstream hydronephrosis without evidence of lithiasis at unenhanced MDCT (not shown) led to performing unenhanced magnetic resonance (MR) urography. Pyelographic (B) and axial T2-weighted images showed smooth tapering of the pelvic ureter (*arrowheads*), without appreciable mural thickening or extrinsic tissue at the coronal T1-weighted image (D), consistent with an iatrogenic stricture

Resulting from previous minor (mucosal) injuries to the urinary tract, late strictures predominantly occur at the distal third of the ureter and are typically depicted by MDCT or MR urography as a tight stricture with upstream hydronephrosis (Fig. 10) or are benign-type smooth tapering without an associated soft-tissue mass (Fig. 11). Since retrograde stenting is frequently unsuccessful, most ureteral injuries are treated by means of nephrostomy with or without a ureteral catheter. Whereas endourological repair of small ureteral fistulae and strictures may be performed in selected cases, most delayed occurrences need deferred reconstructive surgery such as uretero-ureterostomy or uretero-neocystostomy to preserve renal function [5, 33–35].

## Conclusion

Due to the increasing prevalence of urolithiasis and the extensive use of minimally invasive therapies, in hospitals with active urologic practices early or delayed complications following ESWL, PCNL or URS are increasingly encountered. Although uncommon, these occurrences should not be underestimated since they can result in significant morbidity, renal function impairment, occasional medico-legal litigation and death. Appropriate patient selection with consideration of possible contraindications, close monitoring of treated patients for the development of post-procedural complications and prompt use of MDCT imaging are highly recommended [4, 5].

In addition to identifying residual hydronephrosis and stone fragments, unenhanced MDCT is highly useful to diagnose postoperative complications and could be potentially considered in most treated patients [27, 36]. When patients’ complaints, physical examination, laboratory evidence of blood loss or unenhanced MDCT findings suggest a significant complication, prompt CM-enhanced MDCT investigation is warranted as the mainstay technique to provide a quick, comprehensive assessment of renal and ureteral injuries. MDCT consistently allows reliable detection and quantification of retroperitoneal haemorrhage, differentiation from fluid collections or extravasated urine and identification of active bleeding, thus allowing an accurate assessment of the injury’s severity, a correct therapeutic choice and reliable follow-up during conservative treatment [37, 39].
